# Infective Endocarditis and Complications; Surgical Indications and Management: An Integrative Review

**DOI:** 10.3390/jpm16020103

**Published:** 2026-02-09

**Authors:** Daniel Bishev, Michael V. DiCaro, Shudipan Chakraborty, Gregory-Thomas Stanger, Camille Ho, Tahir Tak

**Affiliations:** 1 Kirk Kerkorian School of Medicine, University of Nevada Las Vegas, 1701 West Charleston Blvd., Las Vegas, NV 89135, USA; 2 VA Southern Nevada Healthcare System, 6900 North Pecos Rd., Las Vegas, NV 89086, USA

**Keywords:** infective endocarditis, valve surgery, complications of endocarditis, valvular infections, congestive heart failure

## Abstract

Infective endocarditis (IE) is an infection of the endocardial surface of the heart involving native or prosthetic valves, endocardial structures, or intracardiac devices/leads. Unfortunately, incidence has risen in many settings over recent years. Historically, the incidence has been about 3–10 cases for every 100,000 person-years and was elevated to about 13.8 cases per 100,000 person-years in 2019. Despite advancements in both detection and treatment, mortality remains high, seen with inpatient mortality rates of 18%, along with a 6-month mortality rate of 30%. IE can be a fatal condition if left untreated, in part due to the multiple serious complications that can arise. By anticipating certain complications, clinicians can be better prepared to treat patients with this condition. This article provides an integrative review of the potential complications of IE. These complications vary depending on whether the patient has native or prosthetic valves. There are cardiac, embolic, and immune-complex mediated complications that can occur. Ultimately, IE can lead to multiorgan dysfunction and result in septic shock and disseminated intravascular coagulopathy (DIC). While the mainstay of treatment for IE remains medical, certain cases require surgical intervention. Due to their close relationship, a review of the indications for surgery in the treatment of IE is also presented in this article. By having a general scope of the complications of IE and when to get a surgical consult, clinicians can be better equipped to care for patients with a potentially fatal condition that is becoming increasingly more frequent.

## 1. Introduction

Infective endocarditis (IE) is an infection of the endocardial surface of the heart involving native or prosthetic valves, endocardial structures, or intracardiac devices/leads [1,2]. Unfortunately, incidence has risen in many settings over recent years. Across the world, the number of IE cases and deaths has increased over the last 30 years from 478,000 in 1990 to 1,090,530 in 2019 and from 28,750 in 1990 to 66,320 in 2019 [3]. There is healthcare-associated IE, but there are growing contributions from injection drug use [2,3,4,5]. The number of global incident cases was around 1.09 million, leading to about 66,300 deaths in 2019 [4,6]. Male individuals are more commonly diagnosed with IE than female individuals, particularly older men with a mean age of 67 years, in typical western populations [5]. *S. aureus* now leads as the most common organism in high-income countries, and mortality remains high despite advances in both antibiotics and surgical options [1,2,3,4]. Despite advancements in both detection and treatment, mortality remains high, seen with inpatient mortality rates of 18%, along with a 6-month mortality rate of 30% [7]. IE can be a fatal condition if left untreated, in part due to the multiple serious complications that can arise. By anticipating certain complications, clinicians can be better prepared to treat patients with this condition. The following is a review article about the complications of IE and the indications for surgical intervention.

IE disease patterns differ by substrate: native valve IE, prosthetic valve IE (PVE), and device-related IE (i.e., leads, pockets, or indwelling catheters) [8,9]. Staphylococci (esp. *S. aureus*) predominates in healthcare-associated disease and device/catheter/PVE. Cardiovascular Implantable Electronic Devices (CIEDs) and Transcatheter Aortic Valve Replacement (TAVR) have expanded the spectrum and complexity of IE in both disease process and complications [1,10]. The cardiac complications that can occur are acute valvular regurgitation, acute congestive heart failure, paravalvular abscesses, cardiac aneurysms and intracardiac fistulae, and conduction abnormalities [1,2,10]. There are also embolic complications that arise, such as ischemic and hemorrhagic cerebrovascular events, mycotic aneurysms, pulmonary emboli (in right-sided IE), and systemic emboli (i.e., spleen, kidney, mesentery, etc.) [1,3,10,11]. Immune-complex mediated complications of IE include glomerulonephritis, arthritis, retinal hemorrhages (Roth spots), and Osler nodes. Ultimately, IE can lead to multiorgan dysfunction and result in septic shock and disseminated intravascular coagulopathy (DIC) [1,3,6].

While the mainstay of treatment for IE remains medical, certain cases require surgical intervention. Due to their close relationship, a review of the indications for surgery in the treatment of IE is also presented in this article. Severe valve dysfunction, heart failure, penetrating or peri-annular complications, persistent infections, prevention of systemic embolism, pathogen considerations, cerebral emboli, and prosthetic valve dehiscence are the main indications for surgery in IE [2,3,10]. By having a general scope of the complications of IE and when to get a surgical consult, clinicians can be better equipped to care for patients with a potentially fatal condition that is becoming increasingly more frequent.

## 2. Methods

A literature review was conducted using PubMed/MEDLINE, the Cochrane Library, and PsycINFO to identify peer-reviewed studies relevant to complications of infective endocarditis and indications for valve surgery. A structured search strategy was applied in each database using combinations of controlled vocabulary (e.g., MeSH where applicable) and free-text terms. Search terms included infective endocarditis, valve surgery (or valvular surgery), complications, and review, which were combined with Boolean operators (AND/OR) and adapted to the syntax of each database.

Search results were screened for relevance to the study question through a two-stage process. First, titles and abstracts were reviewed to exclude clearly irrelevant records. Second, potentially eligible articles underwent full-text assessment. Studies were selected based on their relevance to surgical management of IE and its major complications, the clarity and rigor of reported methods, and the applicability of findings to contemporary clinical practice. When multiple publications addressed similar topics, priority was given to higher-quality evidence (e.g., systematic reviews, meta-analyses, randomized trials when available, and well-designed observational cohorts) and to articles with clear definitions of complications and surgical indications.

## 3. Complications

### 3.1. Paravalvular Abscess

Occasionally, IE can spread beyond the valve apparatus itself. Intracardiac abscess occurs as a complication of IE in about 14% of cases [9]. One manifestation of this is paravalvular abscess. Peri-annular extension manifests as thickened, echo lucent, or heterogeneous regions on echocardiography. It often mandates surgery; risk is heightened with aortic valve IE and prosthetic valve endocarditis (PVE) [1,2,3].

Peri-annular extension (PAE) occurs when the infection damages tissue around the valve annulus, leading to abscess, fistula, or valve dehiscence. It complicates about 30% of native and 55% of PVE. Invasive disease is rare in right-sided endocarditis (0.72%) but more common in aortic (65%) and mitral (31%) [12]. Abscesses are often connected with other complications such as fistulas and heart block.

### 3.2. Fistulas

Fistulas related to IE are described as tracts that have been formed as a result of the infectious process. Aorto-cavitary or other intracardiac fistulous tracts can follow abscess; although uncommon, these have a high risk of mortality and typically warrant surgical intervention [3,10]. Congestive heart failure (CHF) is the most critical complication in native and prosthetic valve endocarditis, leading to poor outcomes with medical treatment alone. It strongly predicts better survival with surgery [3]. In infective endocarditis, CHF commonly arises from fistula formation, which creates abnormal shunting between cardiac chambers or vessels, leading to a high cardiac output state [13]. Prompt detection and intervention are critical.

### 3.3. Arrhythmias and Heart Block

Conduction system involvement, such as a new atrioventricular (AV) block, suggests peri-annular extension. It is seen in about 4–8% of cases and is a surgical red flag [1,2,6,14]. Conduction problems are tightly related to the complications. Peri-annular spread of infective endocarditis, accompanied by surrounding pus formation, can cause abscesses and fistulas, resulting in conduction system involvement. This can have multiple presentations ranging from asymptomatic first-degree AV block to complete heart block, potentially resulting in severe and even fatal hemodynamic instability [15]. Although it can resolve after surgery, high-grade AV block is a marker of more advanced disease and generally does not reverse after surgery [1].

### 3.4. Acute Valvular Incompetence and CHF

Acute structural valve injury is a well known and feared complication of IE that can result in severe, acute valvular incompetence, leading to pulmonary edema and possibly even cardiogenic shock [14,16]. This occurs in about one-third of all IE cases [9]. Bacterial invasion of the valve tissue as well as the leaflet support apparatus causes valvular damage such as leaflet perforation, cusp rupture, chordal rupture, or large vegetation-related malcoaptation [17,18]. This causes faster equalization of pressures between cardiac chambers and results in reduced cardiac output. In acute aortic insufficiency (AI), a rapid rise in left ventricular end diastolic pressure (LVEDP) increases cardiac wall tension, thereby reducing coronary perfusion. Pulmonary venous pressures rise rapidly, precipitating sudden pulmonary edema and shock [14,16,17,18]. In severe acute mitral regurgitation (MR), a large regurgitant volume into a normally sized LA generates marked V waves and reduced forward flow, which also causes flash pulmonary edema. In acute MR and AI caused by IE, new onset heart failure is the strongest driver of mortality and is a leading indication for urgent/emergent surgery [1,2,3,4,10,19]. The severity of valvular dysfunction and heart failure are considerations that are made when deciding whether to involve the cardiac surgeons.

### 3.5. Septic Embolic Phenomena

Clinically evident non-central nervous system (CNS) embolization occurs in about 23–33% of cases. The risk correlates with large or mobile vegetations (≥10–15 mm) and cases where the causative organism is determined to be *S. aureus* [1,3]. Meta-analyses show that vegetation length greater than 10 mm confers approximately two-fold higher odds of embolism and increased mortality. Several series highlight even greater risk at thresholds over 15 mm and with marked mobility [20,21].

The embolic hazard is front-loaded, meaning most events occur before or within the first 1–2 weeks after antimicrobial therapy is started. The risk declines thereafter—an observation that underpins recommendations for early surgery in selected high-risk anatomies such as large or mobile vegetations with prior embolism. Preventive splenectomy or routine anticoagulation is not indicated; management focuses on rapid bactericidal therapy and timely surgery when guideline criteria are met and are discussed in more detail below [22].

### 3.6. Pulmonary Complications (Right-Sided IE)

Right-sided IE—classically among people who inject drugs and in patients with intracardiac devices—often presents with recurrent septic pulmonary emboli [1,2,10]. It occurs in about 5 to 10% of all IE cases. The TV is involved in 90% of right-sided IE cases, whereas the pulmonic valve IE may occur concomitantly with TV IE. Isolated pulmonic valve IE is rare and accounts for less than 2% of patients with IE, with only 70 reports of isolated pulmonic valve IE published between 1979 and 2013 [23].

CT imaging typically reveals multiple bilateral nodules (frequently cavitary), wedge-shaped subpleural opacities, and the “feeding vessel” sign. These lesions can progress to lung abscesses, empyema, or rarely pneumothorax, and should prompt aggressive pathogen-directed therapy and source control [1,3,24]. Intravenous antibiotics remain the cornerstone of treatment of right-sided IE, although certain cases may require surgical intervention. Difficult-to-eradicate microorganisms such as *S. aureus* and *P. aeruginosa,* large and persistent vegetations, and severe valvular dysfunction are all indications for surgical intervention [23].

### 3.7. Neurologic Sequelae

Neurologic complications in IE include ischemic and hemorrhagic cerebral vascular events and aneurysms. Stroke occurs in about 17–20% of cases. Hemorrhage and mycotic aneurysms are less common but carry a high risk of morbidity and mortality. Brain and neurovascular imaging help guide the timing of cardiac surgery [1,4,10,25]. Neurologic events are the most frequent extra-cardiac complications of IE [12]. Ischemic strokes predominate as the most common neurologic complications that occur in IE. Systematic imaging demonstrates a high burden of otherwise silent cerebral lesions—MRI studies report acute ischemic lesions in about 60–70% of people, cerebral microbleeds in about 50%, and hemorrhagic lesions in about 25%, even among patients without focal neurologic deficits. These findings influence surgical timing, anticoagulation decisions, and perioperative risk stratification [26,27].

### 3.8. Renal Failure

Acute kidney injury (AKI) and failure can be both pre-renal and intrarenal in IE. Renal dysfunction may result from embolic infarction, immune-mediated GN, sepsis, or nephrotoxic antibiotics used to treat IE. Hemodialysis is required in about 1% of cases in large datasets [3,6]. Common renal lesions identified were localized infarcts in 31% of cases and acute glomerulonephritis in 26% of cases. The most common type of glomerulonephritis was vasculitis, without deposition of immunoproteins in the glomeruli [28]. Half of renal infarcts were due to septic emboli, mostly in patients infected with *S. aureus* [29].

Advanced renal insufficiency due to diffuse glomerulonephritis was associated with both failure of antibiotic therapy to eradicate infection and failure to recover renal function. In patients with diffuse glomerulonephritis and less severe impairment of renal function, antibiotic therapy was successful in achieving bacteriologic cure, and complete recovery of renal function occurred in most cases [28,30,31]. There is a trend towards a higher mortality rate in conjunction with advancing CKD stage [32]. Patients on hemodialysis are at increased risk for IE compared with the general population. The incidence of IE in 1996 was 483 per 100,000 person-years in HD patients, while it was 6.5 per 100,000 person-years in the general US population [33]. This large discrepancy is typically explained by these patients having indwelling catheters with frequent interaction with the medical community [32,33].

### 3.9. Prosthetic Valve Endocarditis with Dehiscence

Dehiscence of a prosthetic aortic valve is an uncommon complication that is reported in 0.1% to 1.3% of patients who undergo aortic valve replacement [34,35]. Transthoracic echocardiography (TTE) has low sensitivity for prosthetic valve dehiscence, with 3D imaging using transesophageal echocardiography (TEE) being recommended for these patients [28,29]. On echocardiography, a rocking motion of the valve is typically accompanied by severe paravalvular regurgitation, often leading to heart failure and typically requiring urgent surgery [2,3]. Three-dimensional (3D) echocardiography is an additional tool that can assist in the diagnosis of dehiscence. Specifically in mitral valve dehiscence, real-time 3D echocardiography provided additional information about the exact anatomic characteristics that assisted in planning the most appropriate corrective procedure [36].

### 3.10. Infective Endocarditis in Patients with CEIDs: Prevention, Diagnosis, and Management

Device-related infective endocarditis, encompassing infections of CIEDs such as pacemakers, implantable cardioverter-defibrillators (ICDs), and cardiac resynchronization therapy devices, represents a rapidly growing subset of IE. This rise parallels the expanding use of intracardiac hardware in aging populations with increasing comorbidity burden. CIED-related infections are associated with substantial morbidity, prolonged hospitalization, recurrent bacteremia, and increased mortality.

Risk stratification is central to both prevention and early diagnosis of device-related IE. Patient-related risk factors consistently associated with higher infection rates include advanced age, chronic kidney disease (particularly end-stage renal disease requiring dialysis), diabetes mellitus, heart failure, chronic corticosteroid or immunosuppressive therapy, malignancy, and a prior history of CIED infection. These comorbidities impair host immune response and wound healing, increasing susceptibility to both pocket and systemic infection. Additionally, patients with poor oral health, chronic skin disease, and frequent healthcare exposure are at increased risk of hematogenous seeding of device leads. Cumulative risk increases substantially when multiple factors coexist, which warrants heightened vigilance and intensified preventive measures in such populations.

Preventive strategies for CIED-related IE are multifaceted and begin prior to device implantation. Pre-procedural optimization includes treatment of active infections, glycemic control in diabetic patients, careful assessment of renal function, and avoidance of unnecessary device implantation when indications are marginal. Strict adherence to sterile technique and appropriate skin antisepsis are foundational measures.

Peri-procedural antibiotic prophylaxis is universally recommended and should be administered within one hour prior to incision. Recent data support the selective use of adjunctive local antimicrobial strategies in high-risk patients. A 10-year propensity-matched analysis demonstrated that gentamicin-impregnated collagen sponges placed in the device pocket were associated with a significant reduction in CIED infection rates without increased adverse events. Such adjunctive measures may be particularly beneficial in patients undergoing generator replacement or those with multiple risk factors.

Post-procedural care focuses on meticulous wound management, early recognition of pocket abnormalities, and patient education regarding signs of infection. Emerging literature also emphasizes the role of antimicrobial stewardship and tailored antibiotic regimens based on local resistance patterns.

Once CIED-related IE is diagnosed, complete removal of all hardware is generally required. Device extraction is typically followed by prolonged pathogen-directed antimicrobial therapy. A unique and clinically challenging aspect of CIED-related IE is the management of arrhythmic risk following device extraction. Pacemaker-dependent patients or those with prior malignant ventricular arrhythmias may require temporary pacing via externalized active-fixation leads or transvenous systems. In patients previously protected by ICD therapy, wearable cardioverter-defibrillators may serve as a bridge until reimplantation is deemed safe. Decisions regarding timing and location of reimplantation must be individualized, balancing infection clearance with arrhythmic risk and often require multidisciplinary input.

### 3.11. Diagnostic Challenges

Despite advances in imaging and microbiologic techniques, the diagnosis of IE remains challenging in a significant subset of patients. Culture-negative IE accounts for approximately 5–25% of cases and may result from prior antibiotic exposure, fastidious organisms (e.g., Coxiella burnetii, Bartonella species), or nonbacterial thrombotic endocarditis. These cases are associated with diagnostic delay, increased complication rates, and worse outcomes.

Echocardiography remains the cornerstone of IE diagnosis; however, its sensitivity is reduced in prosthetic valve endocarditis and device-related IE due to acoustic shadowing and artifact. In these settings, advanced imaging modalities have become increasingly important. Fluorodeoxyglucose positron emission tomography combined with computed tomography (FDG PET-CT) has emerged as a valuable adjunct, particularly for suspected prosthetic valve endocarditis and CIED-related infection, improving diagnostic sensitivity when echocardiographic findings are equivocal. Incorporation of PET-CT findings according to the 2023 ESC guidelines into diagnostic algorithms significantly influences subsequent surgical decision-making. Cardiac CT and PET-CT have been found to be more accurate than echocardiograms in detection of perivalvular and periprosthetic complications and can be used as an adjuvant in detecting extracardiac septic emboli and locating metastatic foci of infection [37].

## 4. Surgical Indications

IE carries substantial short- and long-term mortality, and complications like HF, embolism, and renal injury are common. Complications like these underpin the lower thresholds for early surgery in certain clinical scenarios. Surgery is done for IE in about 40–50% of cases [7]. In-hospital and short-term mortality hovers around 20% and long-term mortality approaches 37–45% [3,6]. For this reason, clinical decisions need to be made promptly to maximize the chances for successful treatment.

Cardiac surgery in IE is lifesaving when there is (1) heart failure from valve destruction, (2) uncontrolled/penetrating infection, or (3) a need to prevent further embolic events in high-risk anatomy. Decisions should be made early by a multidisciplinary IE team, which includes cardiologists, cardiac surgeons, infectious disease specialists, neurologists, and imaging specialists. The need for a collaborative approach and quick decision-making is critical because progression can be rapid, and mortality remains high despite appropriate antibiotic therapy alone.

The need for surgery in IE can be defined in three categories, as emergent, urgent, or delayed/elective. The most recent (ESC 2023) guidelines and supporting literature organize the timing of surgery as emergent (hours), urgent (days), or delayed/elective (during index hospitalization, typically within the first 1–2 weeks [1,2,10]. Below are the most common indications for surgery in the treatment of IE. Table 1 lists the absolute and relative indications for surgical intervention.

The timing of surgical intervention has been debated in the past. To date, the timing remains unclear between guidelines due to limited data and interpretation of the clinical trials to date. Surgery by the latest ESC 2023 guidelines recommend emergent surgery within 24–48 h in patients with NYHA class III or IV symptoms, severe valvular regurgitation or destructive valvular lesions leading to hemodynamic instability and for embolic phenomenon prevention. Urgent surgery is recommended within days for severe valve regurgitation or destructive valvular lesions without instability, refractory infections, or large (>10 mm) lesions. Delayed surgery is recommended in 1–2 weeks for stable patients after an initial antibiotic course to reduce inflammation. A major retrospective review looking at 29,270 cases of IE between 2016 and 2020 who received surgical treatment for left-sided IE found that there was a benefit of surgical intervention within the first seven days of hospitalization. The trial found a statistically significant reduction in the likelihood of acute CVA, peripheral septic emboli, and intracranial or intraspinal abscess. However, it was not associated with an overall decrease in all-cause in-hospital mortality [38].

All treatment strategies and decisions should be performed on a case-by-case basis regarding close monitoring and identification of clinical complications. As outlined above in Table 1, certain signs and symptoms recommend an early approach to surgery. However, in patients with symptomatic or moderate to large territory ischemia strokes there is an added risk or perioperative hemorrhagic transformation in the setting of high dose anticoagulation provided during cardiopulmonary bypass. In these cases, a waiting period of 2 to 4 weeks after the ischemic event is undertaken prior to proceeding with surgery [39].

### 4.1. Severe Valve Dysfunction

Severe valve dysfunction occurs in about one-third of all IE cases [9]. HF from acute MR or AI rarely stabilizes without operative source control [1]. Echocardiographic features that track with poorer outcomes and earlier surgery include large, mobile vegetations and severe regurgitation. Acute severe aortic or mitral regurgitation causing pulmonary edema, cardiogenic shock, or persistent NYHA III–IV symptoms demand emergent or urgent surgery; medically refractory HF is the strongest predictor of benefit from operation [2,3]. Elective surgery can be considered for moderate regurgitation with progressive dilation or dysfunction, even if symptoms transiently improve with therapy [1,2,10,40].

Right-sided IE occurs largely due to injection drug use, and most cases respond to antibiotics alone. Surgery is considered for persistent bacteremia, recurrent septic pulmonary emboli with large tricuspid vegetations (>20 mm), or severe tricuspid regurgitation causing right-sided HF. Valve-sparing or vegetation-debulking strategies are preferred when feasible [1,40].

### 4.2. Penetrating or Peri-Annular Complications

Occasionally, the infection can spread to the surrounding cardiac structures, which can also be an indication for surgery. Penetrating or peri-annular complications include abscesses, fistulae, or aneurysms [2]. Urgent surgery is required when there is an annular or aortic root abscess, a mycotic aneurysm with intracardiac communication, valve leaflet perforation with hemodynamic compromise, prosthetic valve dehiscence, or new AV block suggesting extension into the conduction system [2,3,10]. Surgical emergencies such as fistulas to intracardiac chambers or pericardial structures are surgical emergencies [1,2,10,40]. These penetrating infections rarely sterilize with antibiotics alone and carry high risks of rupture, persistent sepsis, and death. For this reason, it is important to get surgical evaluation early when a penetrating infection is suspected [1,3,39].

**Figure 1:**
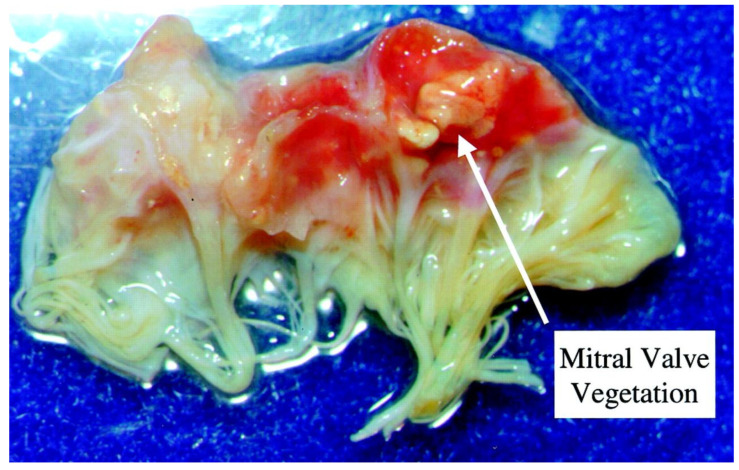
This figure shows one portion (called a leaflet) of the mitral valve of the heart. The valve has been excised surgically in the course of treating endocarditis. There is a large mass or vegetation on the valve, and it is surrounded by bleeding into the valve tissue that has resulted from valve damage [42].

**Figure 2:**
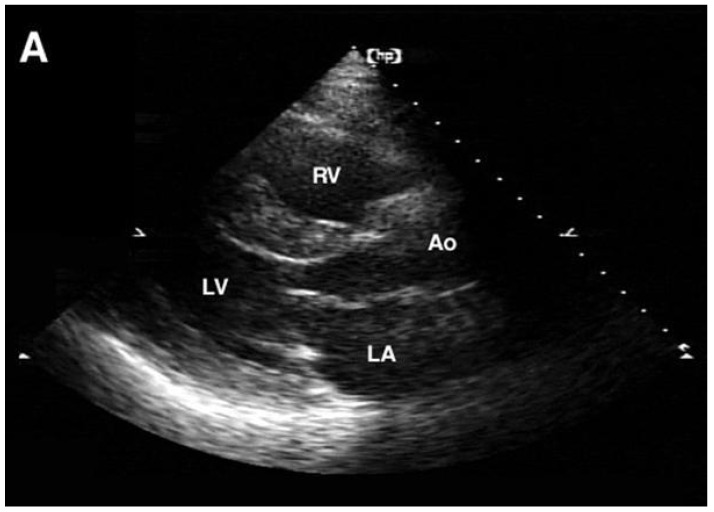

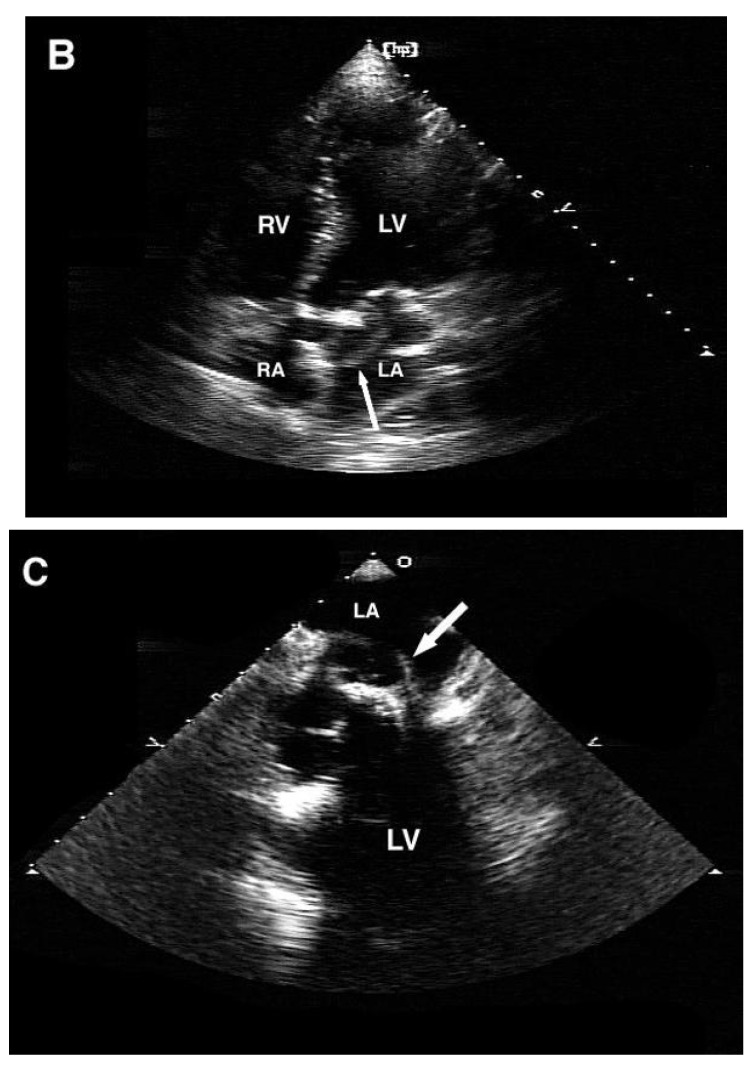

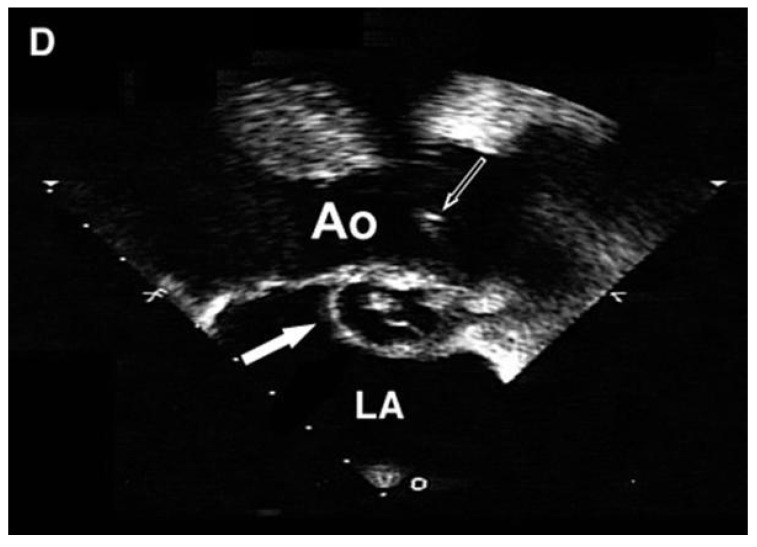
(**A**,**B**): Transthoracic parasternal and apical two chamber views showing the pseudoaneurysm and its proximity to the prosthetic aortic valve (see arrow). (**C**,**D**): Transesophageal echocardiography delineating the pseudoaneurysm and encroachment of the MAIVF (indicated by bold arrow). The aortic valve is indicated by the open arrow. MAIVF: mitral-aortic intervalvular fibrosa; LV: left ventricle; RV: right ventricle; LA: left atrium; RA: right atrium; Ao: aorta [43].

**Figure 3:**
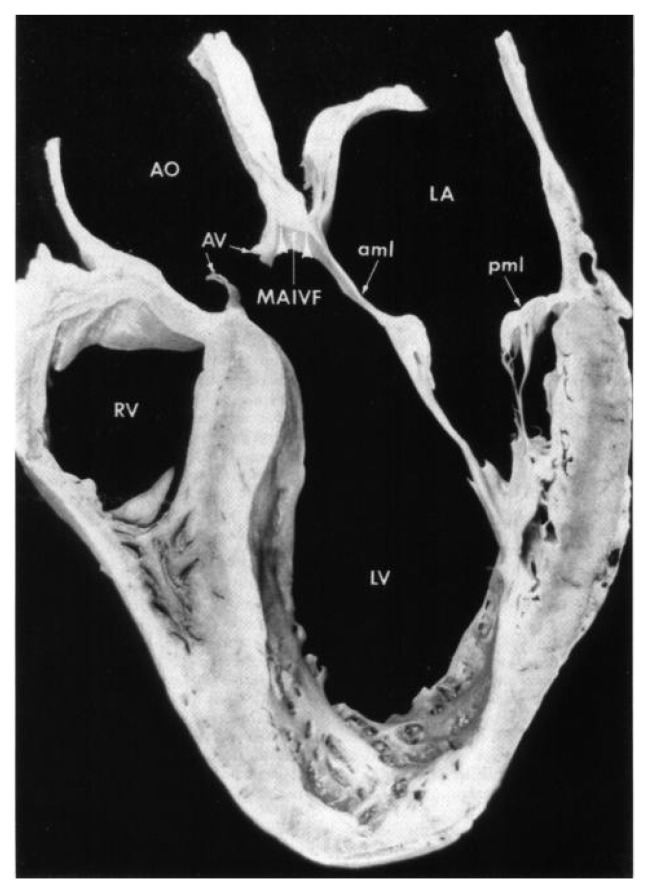
General necropsy image of the heart (not from our patient) showing the relation of the aortic valve (AV), annulus, and root (AO) to the subaortic structures, including the MAIVF. The anterior mitral leaflet becomes continuous with the posterior aortic root. MAIVF: mitral-aortic intervalvular fibrosa; aml: anterior mitral leaflet; pml: posterior mitral leaflet; LV: left ventricle; RV: right ventricle; LA: left atrium; Obtained with permission from Tak T. Pseudoaneurysm of mitral-aortic intervalvular fibrosa [43].

The mitral-aortic intervalvular fibrosa (MAIVF) is a thin avascular fibrous tissue connecting the anterior mitral valve leaflet and the aortic annulus, most specifically by the left and non-coronary cusps. The periaortic spread of the infection can penetrate deep tissues, such as the MAIVF. The proximity of the mitral valve to the MAIVF can be seen in Figure 1, demonstrating a large vegetation of the mitral valve. If this structure gets affected, it initially causes MAIVF thickening, which can progress to the formation of an abscess and, subsequently, a pseudoaneurysm [41]. There was a case report published in 2003 about a woman who developed a pseudoaneurysm of the MAIVF. Figure 2 and Figure 3 were obtained from this case report. Figure 2 shows transthoracic and transesophageal views of the pseudoaneurysm encroachment on the MAIVF and Figure 3 is a necropsy image of the heart obtained from that case report [42].

### 4.3. Persistent Infection

Persistent infection is another indication for consideration of early surgery. Ongoing bacteremia signals uncontrolled intracardiac infection, such as a hidden abscess or prosthetic involvement, where cure typically requires debridement or reconstruction [1,2]. It is defined as continued infection despite appropriate antimicrobial therapy. Early surgery is recommended for persistent bacteremia or fevers despite adequate, organism-directed IV therapy (often cited at about 5–7 days in practice), after excluding an undrained extracardiac focus [2,10]. This is particularly pressing for S. aureus, fungal IE, and multidrug-resistant organisms [1,2,4].

### 4.4. Prosthetic Valve Dysfunction or Dehiscence

Any prosthetic valve endocarditis (PVE) that leads to valve dysfunction is an indication for early or urgent surgery [2,3]. TAVR-IE has high rates of paravalvular leak and abscess, and CIED-related IE has lead involvement for which management typically requires explant and debridement concurrently alongside antibiotics [4,10]. Severe regurgitation, outlet obstruction from vegetations, paravalvular leak, or abscess are all indications for surgery. Surgery is often required even without overt HF because PVE frequently involves the sewing ring and annulus [1,2,40].

PVE accounts for 30% of cases in the EURO-ENDO Registry, 25% in the 2008 French registry, and 21% in the International Collaboration on Endocarditis Prospective Cohort Study reported in 2009 [7]. PVE is a relatively common complication of valve replacement, occurring at a rate of 0.3% to 1.2% per patient-year. This means that about 3% to 6% of individuals who receive a prosthetic valve will develop IE as a complication within the first 5 years following implantation [7].

### 4.5. Prevention of Systemic Embolism

There are certain criteria for the size and location of vegetations that are considered high-risk for embolization. Surgical intervention is performed for this reason in about 40–50% of cases [7]. As such, they qualify for surgical intervention. Embolic risk is front-loaded (highest in the first 1–2 weeks of therapy) and falls after effective antibiotics; large, mobile mitral vegetations carry the greatest risk; thus, in certain situations, operating earlier can improve outcomes [1,40].

Left-sided IE with large vegetations is an indication for early surgery, described as follows:

- severe AI/MR with residual vegetation > 10 mm;
- recurrent emboli;
- embolism plus > 10 mm vegetation;
- vegetation > 15 mm despite appropriate antibiotics [2,3].

For both left and right-sided IE:

(a) When vegetation > 10 mm with severe aortic/mitral regurgitation or prior embolic event, urgent surgery is reasonable;
(b) Vegetation > 15 mm without embolism or severe regurgitation, consider early surgery;
(c) Recurrent emboli despite appropriate antibiotics is an indication for urgent surgery [1,2,40].

### 4.6. Pathogen Considerations

There are certain organisms and clinical situations where surgical intervention may be needed solely based on the specific pathogen. Early surgery is indicated for fungal IE and for highly resistant pathogens such as MRSA or enterococci. Early surgery can be considered for persistent *S. aureus* bacteremia, given the propensity for abscess and embolic complications [1,2,4].

### 4.7. Cerebral Emboli

An ischemic stroke is not a contraindication to early surgery once hemorrhage is excluded and neurologic status is stable; delaying surgery risks further emboli and HF progression. In intracranial hemorrhage or very large territorial infarcts with mass effect, many centers defer surgery for about 4 weeks if feasible [1,2,10].

## 5. Conclusions

IE is becoming an increasingly common disease being faced by the medical community due to an aging population in the advent of an increased number of intracardiac cardiac device implantations. It has high morbidity and mortality if prompt and proper therapy is not given. Medical management is sufficient for most cases, but others may require surgical intervention. Due to the various possible clinical trajectories, every patient should have a personalized approach depending on patient presentation.

The wide range of possible clinical presentations reinforce the need for clinicians to be well versed in potential complications to maximize detection, start the correct interventions, and involve the appropriate specialists promptly. Early multidisciplinary evaluation, evidence-based surgical triggers, and tailored imaging strategies can help get the proper treatment in a timelier manner and ultimately provide better care for a potentially fatal condition [1,2,3,4,6,10]. When IE is diagnosed, prompt treatment and vigilant monitoring of complications with the use of echocardiogram and adjuvant imaging modalities are quintessential for optimal patient outcomes.

## Figures and Tables

**Table 1:** List of absolute and relative indications for surgical intervention as therapy for infective endocarditis.

Indications for Surgical Therapy	
Absolute	Relative
CHF refractory to medical therapy Persistent bacteremia despite targeted antibiotics At least one significant embolic event Inadequate antibacterial therapy (as in fungal IE) Uncontrolled bacteremia (despite antibiotic treatment) Significant valve dysfunction demonstrated by echocardiography/Doppler Mycotic aneurysm(s) Early PVE and some cases of late PVE Evidence of extension of infection (heart block, perivalvular or myocardial abscess, or fistula)	Staph aureus infection with a large vegetation size of >10 mm Relapses after appropriate antimicrobial therapy

## Data Availability

No new data were created or analyzed in this study. Data sharing is not applicable to this article.
